# Single-cell whole-genome sequencing, haplotype analysis in prenatal diagnosis of monogenic diseases

**DOI:** 10.26508/lsa.202201761

**Published:** 2023-02-21

**Authors:** Liang Chang, Haining Jiao, Jiucheng Chen, Guanlin Wu, Ping Liu, Rong Li, Jianying Guo, Wenqing Long, Xiaojian Tang, Bingjie Lu, Haibin Xu, Han Wu

**Affiliations:** 1 Center for Reproductive Medicine, Department of Obstetrics and Gynecology, Peking University Third Hospital , Beijing, China; 2 National Clinical Research Center for Obstetrics and Gynecology (Peking University Third Hospital) , Beijing, China; 3 Key Laboratory of Assisted Reproduction (Peking University) , Ministry of Education, Beijing, China; 4 Beijing Key Laboratory of Reproductive Endocrinology and Assisted Reproductive Technology, Beijing, China; 5 Department of Obstetrics and Gynecology, Rui-Jin Hospital, Shanghai Jiao Tong University School of Medicine, Shanghai, China; 6 Unimed Biotech (Shanghai) Co., Ltd., Shanghai, China

## Abstract

Monogenic inherited diseases are common causes of congenital disabilities, leading to severe economic and mental burdens on affected families. In our previous study, we demonstrated the validity of cell-based noninvasive prenatal testing (cbNIPT) in prenatal diagnosis by single-cell targeted sequencing. The present research further explored the feasibility of single-cell whole-genome sequencing (WGS) and haplotype analysis of various monogenic diseases with cbNIPT. Four families were recruited: one with inherited deafness, one with hemophilia, one with large vestibular aqueduct syndrome (LVAS), and one with no disease. Circulating trophoblast cells (cTBs) were obtained from maternal blood and analyzed by single-cell 15X WGS. Haplotype analysis showed that CFC178 (deafness family), CFC616 (hemophilia family), and CFC111 (LVAS family) inherited haplotypes from paternal and/or maternal pathogenic loci. Amniotic fluid or fetal villi samples from the deafness and hemophilia families confirmed these results. WGS performed better than targeted sequencing in genome coverage, allele dropout (ADO), and false-positive (FP) ratios. Our findings suggest that cbNIPT by WGS and haplotype analysis have great potential for use in prenatally diagnosing various monogenic diseases.

## Abstract (precis)

cbNIPT by WGS and haplotype analysis predict the inherited genotypes for families with monogenic diseases and provide a potentially better solution for prenatal diagnosis of monogenic diseases.

## Introduction

Monogenic disorders typically result from a single gene lesion. Although most individual monogenic diseases are rare, combined, they affect 10 in 1,000 births (WHO | human genomics global health, 2019), representing a substantial threat to human health. As of 12 July, 2022, the Online Mendelian Inheritance in Man (OMIM) database (https://www.omim.org/statistics/geneMap) has reported 6,134 monogenic diseases with known pathogenetic mutations, involving 4,288 genes. Therefore, it is necessary to monitor these genetic risks through prenatal examinations to better manage potential congenital abnormalities caused by genetic diseases. The first prenatal diagnostic tests for genetic diseases were developed 60 yr ago. Chorionic villus sampling and amniocentesis coupled with advanced cytogenetics and molecular diagnosis can now detect most diseases with known genetic causes (Steele & Breg, 1966; Nadler & Gerbie, 1970; Simoni et al, 1983; Levy & Stosic, 2019). Unfortunately, these prenatal tests are invasive and pose a potential risk to the fetus; gestational loss occurs in <1% of individuals undergoing such tests (Simpson, 2012; Alfirevic et al, 2017; Salomon et al, 2019). Moreover, in some underdeveloped countries and regions, it is difficult to perform invasive examinations regularly because of the lack of medical resources.

Noninvasive prenatal testing (NIPT), an important risk-free prenatal examination with improving accuracy, is becoming increasingly popular in clinical practice (Brady et al, 2016). Remarkable achievements have recently been made in detecting prenatal diseases with NIPT based on cell-free fetal DNA (cffDNA) isolated from maternal peripheral blood (Rabinowitz & Shomron, 2020). However, various risk factors in cffDNA-based NIPT, including a limited detection rate for chromosome structural abnormalities and single gene mutations, the low concentration and instability of cffDNA in maternal blood, fetal/placental mosaicism, and maternal chromosome abnormalities, can lead to inaccurate test results (Spits & Sermon, 2009; Beaudet, 2016; Rabinowitz & Shomron, 2020). In contrast to cffDNA, the rare circulating fetal cells in the maternal blood, mainly circulating trophoblast cells (cTBs) and fetal nucleated red blood cells (fNRBC), represent unique sources of fetal DNA without maternal interference (Zipursky et al, 1959; Walknowska et al, 1969; Herzenberg et al, 1979; Beaudet, 2016; Singh et al, 2017). Given the recent progress in single-cell genomics, researchers have explored cell-based NIPT (cbNIPT) because of the advantage of studying pure and intact fetal genetic material from fetal cells. It has been demonstrated that fetal cells captured from maternal blood can be used for subsequent sequencing to detect chromosomal (Breman et al, 2016) and subchromosomal (Kolvraa et al, 2016; Hou et al, 2017; Vossaert et al, 2019) abnormalities after DNA extraction and amplification.

Our previous study confirmed that the targeted sequencing of a 67-gene panel combined with a haplotype analysis could detect monogenic diseases (e.g., congenital deafness and ichthyosis) from individual cTBs captured from the maternal peripheral blood (Chang et al, 2021). Whole-genome sequencing (WGS) has certain advantages over targeted NGS for detecting mutations beyond the targeted regions and complex structural variations (Palmer et al, 2021). However, few studies on WGS applications at the single-cell level and performance surveys comparing WGS with other sequencing methods have been conducted in clinical settings. Because of the limited genetic material, single-cell DNA sequencing usually requires whole-genome amplification (WGA), leading to biases in the sequencing data, such as non-uniformity of genome coverage and high allele dropout rates (Zhang et al, 2015; Volozonoka et al, 2022). These intrinsic limitations complicate downstream analyses, including genomic variant detection (Satas & Raphael, 2018), and prevent direct mutation analysis. Haplotype analysis could be beneficial in this scenario (Clark, 1990). Common haplotype analyses mainly use population data or multiple cells for phasing (Kumar et al, 2015; Guo et al, 2018), but when the number of cells is small, and there are no population data, only relative phasing can be conducted using DNA from the father, mother, and proband. Whether single-cell–based WGS combined with haplotype analysis can be used for clinical diagnosis deserves further investigation.

In this study, we verified the feasibility and scope of single cTB WGS combined with haplotype analysis for examining monogenic diseases inherited from the parents using cTBs and samples from affected family members including the proband, providing the first insight into the potential application of WGS-based cbNIPT in prenatal diagnostics.

## Results

### Clinical information of recruited families

**Figure S1.**
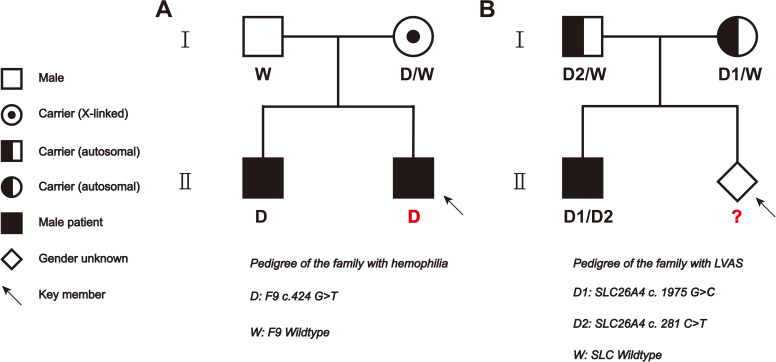
Pedigrees of the tested hemophilia and LVAS families. **(A)** In the hemophilia family, the proband (child) carries a hemizygous mutation of ChrX:*F9* gene c.424G>T from the mother. **(B)** In the LVAS family, the proband (child) carries compound heterozygous mutations of Chr7:*SLC26A4* gene c.1975G>C and c.281C>T from the mother and father, respectively.

A total of four families (one family with deafness, one with hemophilia, one with large vestibular aqueduct syndrome [LVAS], and one healthy control) were included in this study. Except in the deafness family, described in the study by Chang et al (2021), all of the women conceived naturally. In the hemophilia family (Fig S1A), the proband (child) carries a hemizygous mutation of *F9* gene c.424G>T on ChrX, which was inherited from the mother. In the LVAS family (Fig S1B), the proband (child) carries compound heterozygous mutations of *SLC26A4* gene c.1975G>C and c.281C>T on Chr7; c.281C>T was inherited from the father and c.1975G>C from the mother. Details of all the included families are listed in Table 1.

**Table 1.** Clinical and molecular diagnostic information of the four recruited families.

Family	Member	Gender	Age (yr)	Height (cm)	Body weight (kg)	Fertilization method	Pregnancy history	Sample	Type	Genes	Pathogenic loci
Deafness	Mother	Female	36	156	66	In vitro fertilization	Previous pregnancy: 3; spontaneous abortion: 0; full term birth: 0; number of children: 1; artificial abortion: 1	Blood	gDNA	*GJB2*	Chr13:c.299_300delAT, heterozygote
								cTBs-CFC178	WGA	*GJB2*	Chr13:c.235delC, heterozygote
								Amniotic	gDNA	*GJB2*	Chr13:c.235delC, heterozygote
	Father	Male	41	171	76			Blood	gDNA	*GJB2*	Chr13:c.235delC, heterozygote
	Proband	Male	11	154	42			Blood	gDNA	*GJB2*	Chr13:c.299_300delAT, heterozygote
Hemophilia	Mother	Female	34	162	60	Natural	Previous pregnancy: 1; spontaneous abortion: 0; full term birth: 1; number of children: 1; artificial abortion: 0	Blood	gDNA	*F9*	ChrX:c.424G>T, heterozygote
								cTBs-CFC616	WGA	*F9*	ChrX:c.424G>T, hemizygote
								Fetal villi	gDNA	*F9*	ChrX:c.424G>T, hemizygote
	Father	Male	39	165	72			Blood	gDNA	*F9*	Wildtype
	Proband	Male	5	112	18			Blood	gDNA	*F9*	ChrX:c.424G>T, hemizygote
LVAS	Mother	Female	28	Unknown	Unknown	Natural	Previous pregnancy: 4; spontaneous abortion: 0; full term birth: 1; number of children: 1; artificial abortion: 2	Blood	gDNA	*SLC26A4*	Chr7:c.1975G>C, heterozygote
								cTBs-CFC111	WGA	*SLC26A4*	Chr7:c.281C>T, heterozygote
	Father	Male	27	Unknown	Unknown			Blood	gDNA	*SLC26A4*	Chr7:c.281C>T, heterozygote
	Proband	Male	5	Unknown	Unknown			Blood	gDNA	*SLC26A4*	Chr7:c.281C>T, heterozygote Chr7:c.1975G>C, heterozygote
Health	Mother	Female	29	Unknown	Unknown	natural	Previous pregnancy: 0; spontaneous abortion: 0; full term birth: 0; number of children: 0; artificial abortion: 0	Blood	gDNA		
								cTBs-CFC518	WGA		
								cTBs-CFC2282	WGA		
	Father	Male	Unknown	Unknown	Unknown			Blood	gDNA		
	Fetus	Unknown	Unknown	Unknown	Unknown			Saliva	gDNA		

### Capture and confirmation of cTBs

**Figure S2.**
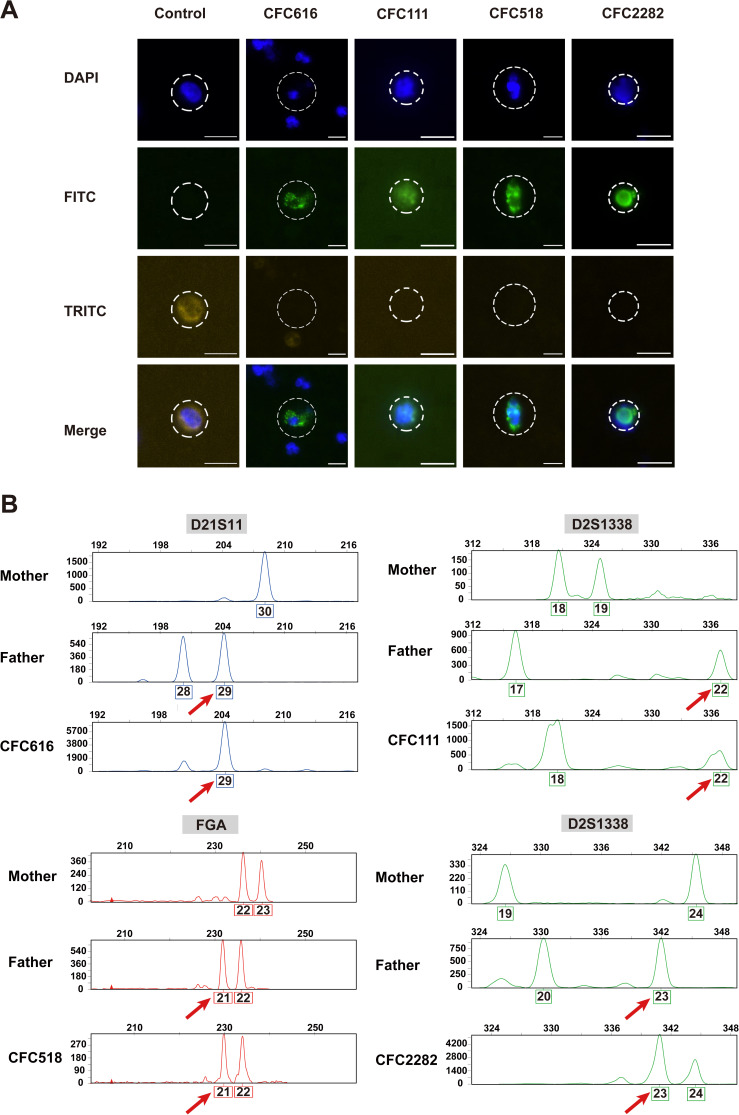
Cell staining images and single-cell STR analysis of captured circulating trophoblast cells. **(A)** Immunofluorescence images of candidate fetal trophoblast cells. Cells were stained with FITC-anti-CK (green), TRITC-anti-CD45 (yellow), and DAPI (blue). Circles indicate the candidate trophoblast cells selected for downstream analyses. **(B)** STR analysis of WGA products from single candidate cTBs and the genomic DNA from paired parents. Cells with multiple paternal alleles were scored as cTBs (arrows and Tables S1–S3). Scale bar is 10 μm.

In all cases, the peripheral blood of pregnant women was subjected to procedures to capture cTBs (see the Materials and Methods section). The deafness family had compound heterozygous mutations in the Chr13:*GJB2* gene (NM_004004.5; c.235delC [p.Leu79Cysfs] and c.299_300delAT [p.His100Argfs]); the details regarding cTB isolation (CFC178) and STR identification in this family are described in a previous study (Chang et al, 2021). After manual confirmation of candidate cells, one or two top candidate cTBs were chosen from the hemophilia, LVAS, and healthy families (Fig S2A). Cell CFC616 was obtained from the family with hemophilia. Cell CFC111 was obtained from the family with LVAS. Cells CFC518 and CFC2282 were isolated from the peripheral blood of pregnant woman from the healthy family. A white blood cell was stained as the control. The candidate trophoblast cells were successfully obtained for the subsequent single-cell analysis.

After single-cell WGA, STR analysis was performed to confirm the origin of candidate cTBs. Representative paternal-specific alleles are shown in Fig S2B, and the data indicated that CFC616, CFC111, CFC518, and CFC2282 were cTBs. In CFC616 from the peripheral blood of a patient carrying hemophilia, 75% (12 out of 16) STR loci were successfully detected and five paternal-specific alleles were identified (Table S1). In the CFC111 cell from the LVAS family, the detection rate of STR loci was 43.75% (7 out of 16), and three paternal-specific alleles were identified (Table S2), confirming the cell’s fetal origin. CFC518 and CFC2282 were isolated from the healthy family, and the detection rates of STR loci were 87.50% (14 out of 16) and 81.25% (13 out of 16), respectively; six and five paternal-specific alleles were identified, respectively (Table S3). Not all STR loci can be identified from single-cell WGA products, likely because of allele dropout (ADO) or PCR failure. Overall, we successfully isolated cTBs from all families with monogenic diseases and the healthy family.

Table S1 STR genotyping results of 16 loci in CFC616 from the hemophilia family.

Table S2 STR genotyping results of 16 loci in CFC111 from the LVAS family.

Table S3 STR genotyping results of 16 loci in CFC518 and CFC2282 from the healthy family.

### Sequencing depth test in the healthy family

**Figure 1.**
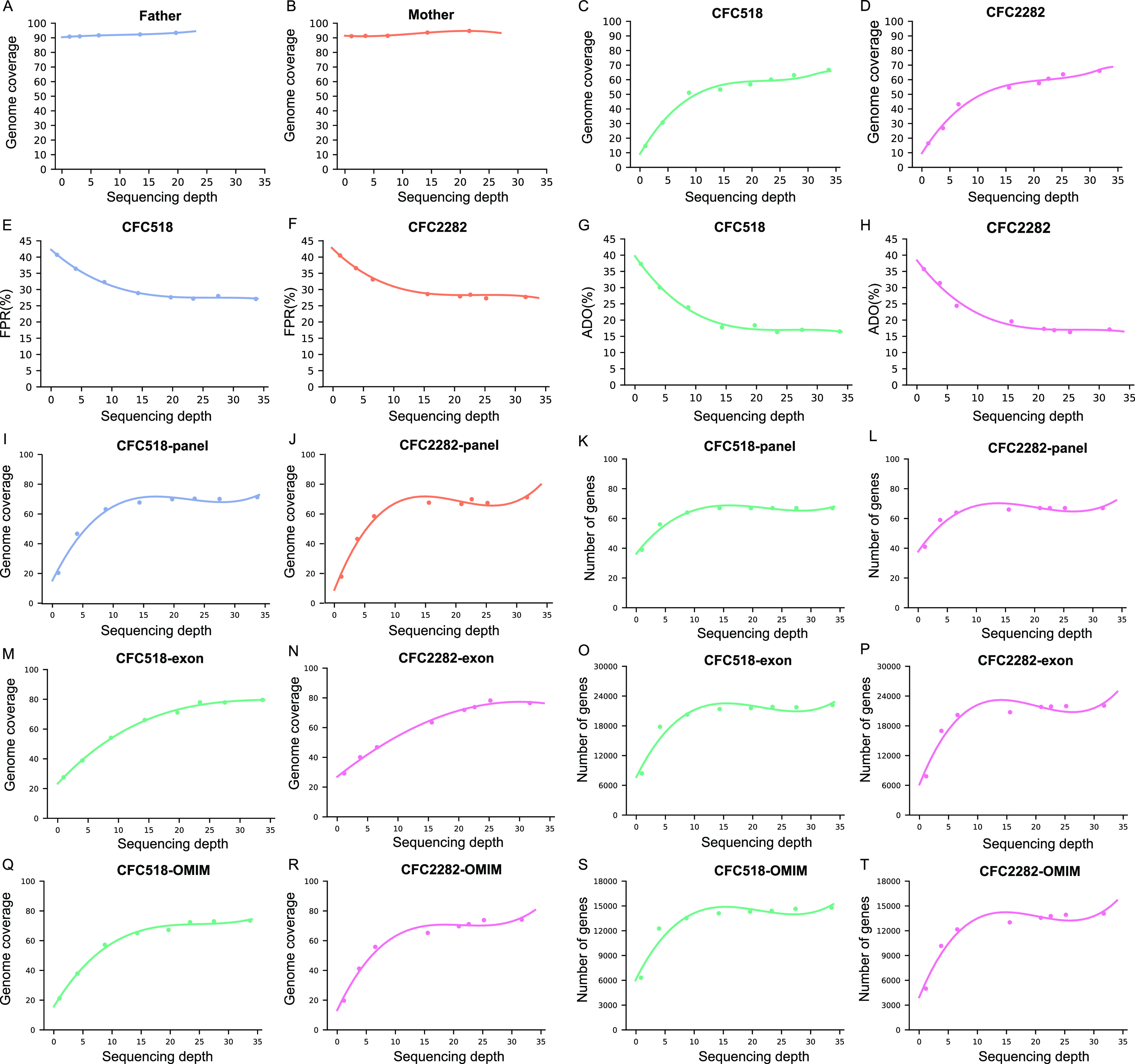
Sequencing depth test in the healthy family. **(A, B)** Genome coverage of different sequencing depths for the father (A) and the mother (B) in the healthy family. **(C, D)** Genome coverage of different sequencing depths for CFC518 (C) or CFC2282 (D). **(E, F)** FPR for CFC518 (E) and CFC2282 (F). **(G, H)** ADO of CFC518 (G) or CFC2282 (H). **(I, J)** Genome coverage of the regions corresponding to the 67-gene panel from WGS of CFC518 (I) or CFC2282 (J). **(K, L)** Number of covered 67-gene panel genes in the WGS of CFC518 (K) or CFC2282 (L). **(M, N)** Genome coverage of the whole-exon region in the WGS of CFC518 (M) or CFC2282 (N). **(O, P)** Number of covered whole-exon region genes in the WGS of CFC518 (O) or CFC2282 (P). **(Q, R)** Genome coverage of the OMIM gene panel in the WGS of CFC518 (Q) or CFC2282 (R). **(S, T)** Number of covered OMIM genes in the WGS of CFC518 (S) or CFC2282 (T).

WGS data from healthy family’s genomic DNAs (gDNAs) and single cells were randomly subsampled at various depths to test the sequencing depth required for appropriate downstream analysis (see the Materials and Methods section). In the WGS data from gDNAs, the genome coverage remained stable at over 90% (Fig 1A and B and Table S4). In the captured cTBs CFC518 and CFC2282, the genome coverage of their single-cell WGS data increased rapidly until the sequencing depth exceeded 15X (Fig 1C and D and Table S4). In addition, when the sequencing depth was lower than 15X, the false-positive (FP) ratios and ADO of CFC518 and CFC2282 decreased with the increase in the sequencing depth (Fig 1E–H and Table S4). Finally, the genome coverage and the number of covered genes in the targeted regions (67-gene panel, whole-exome region, and OMIM gene panel region) by WGS suggested that the coverage of CFC518 and CFC2282 increased linearly with the sequencing depth up to 15–20X (Fig 1I–T and Table S4). These results suggest that a ∼15X sequencing depth is adequate for WGS of gDNA and single-cell WGA products in terms of genome coverage, FDR, and ADO. Therefore, in the subsequent analysis of families with monogenic disease, WGS of individual samples was conducted at a sequencing depth of ∼15X.

Table S4 Gradient simulation of WGS sequencing depth for the healthy family.

### Single-cell haplotype phasing

**Figure 2.**
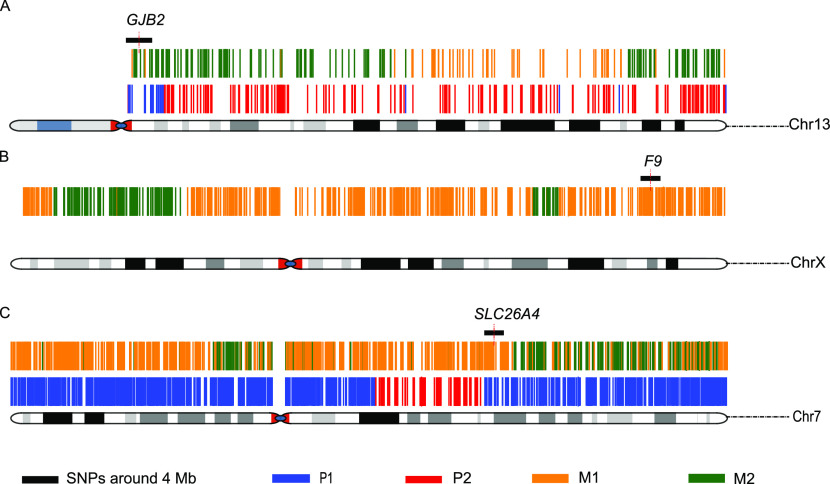
Haplotype inheritance surrounding disease-causing genes in the three monogenic disease families. **(A)** Deafness family with the haplotype P1/M2 in Chr13:*GJB2*. **(B)** Hemophilia family with the haplotype M1 in ChrX:*F9*. **(C)** LVAS family with the haplotypes P1/M1 in Chr7:*SLC26A4*.

The deafness and hemophilia families, unlike the LVAS family, had paired amniotic or fetal villi samples available as references; thus, we could use the ADO and FPR to evaluate the sequencing quality. The FPR (deafness: 30.2%, hemophilia: 26.9%) and ADO (deafness: 17.3%, hemophilia: 18.5%) indicated that the sequencing quality of single cells was compromised because of technical limitations; thus, direct SNP detection may not reliably determine whether single cells carry pathogenic mutations. Therefore, haplotype analysis was required for further analysis. In the deafness family, CFC178 carried the pathogenic haploid P1 (10 upstream key SNPs, three downstream key SNPs) from the father and the nonpathogenic haploid M2 (five upstream key SNPs, five downstream key SNPs) from the mother (Fig 2A and Table S5), consistent with the results from the paired amniotic fluid. Because the causative gene F9 in the hemophilia family was inherited on the X chromosome and the sex of the CFC616 single cell was male, we only needed to consider whether the cell carried the causative gene from the mother. The results (Fig 2B and Table S6) suggested that CFC616 carried the haploid M1 (six upstream key SNPs, seven downstream key SNPs) of the maternal pathogenic loci, consistent with the fetal villi sample. The haplotype analysis of the LVAS family (Fig 2C and Table S7) revealed that CFC111 carried both the paternal and maternal pathogenic chromatid P1 (nine upstream key SNPs, 26 downstream key SNPs) and M1 (23 upstream key SNPs, seven downstream key SNPs). These data suggest that ∼15X WGS per single cell is sufficient for downstream haplotype analysis to accurately predict the heredity of monogenic diseases.

Table S5 Single-nucleotide polymorphisms used to construct fetal haplotypes in the deafness family (P1/M2).

Table S6 Single-nucleotide polymorphisms used to construct fetal haplotypes in the hemophilia family (M1).

Table S7 Single-nucleotide polymorphisms used to construct fetal haplotypes in the LVAS family (P1/M1).

### Comparison of WGS and targeted sequencing

For each single cell from the three families with monogenic diseases (CFC178 from the deafness family, CFC616 from the hemophilia family, and CFC111 from the LVAS family), ∼200X targeted sequencing was performed in addition to 10–15X WGS from the same cell. As a result, we could directly compare the performance of the two methods for investigating the same set of disease-causing genes at the single-cell level (Table 2). In the 67-gene panel region, the genome coverages from the single-cell WGS of the deafness, hemophilia, and LVAS families were 69.3%, 64.8%, and 68.7%, respectively. However, the corresponding coverages using the targeted sequencing method were 60.3%, 56.9%, and 63.1%, respectively. In the targeted sequencing of the 67 genes, the numbers of genes covered were 62, 59, and 62 in the three disease families. Among them, 58, 59, and 61 genes could be used for the haplotype analysis. By using WGS, 65, 65, and 66 of the 67 genes were covered in the three disease families, and all these genes met the requirements for the haplotype analysis. Overall, these results indicate that the WGS method outperformed targeted sequencing in the 67-gene region.

**Table 2.** Sequencing quality assessment of WGS and targeted sequencing in single cells.

Disease		Deafness	Hemophilia	LVAS	Health	
Sample		cTBs-CFC178	cTBs-CFC616	cTBs-CFC111	cTBs-CFC518	cTBs-CFC2282
Genome coverage (%)	WGS	56.8	51.9	57.7	53.3	54.6
Average depth	WGS	14.1	8.4	16.04	14.32	15.56
	Targeted sequencing	114	88.4	110.21	103.8	96.9
Coverage (%) of the 67-gene panel region	WGS	69.3	64.81	68.7	67.7	67.6
	Targeted sequencing	60.31	56.9	63.1	59.5	62.2
Number of covered 67-gene panel	WGS	65	65	66	67	66
	Targeted sequencing	62	59	62	62	63
Number of available 67-gene panel genes for haplotyping	WGS	65	65	66		
	Targeted sequencing	58	59	61		
Coverage (%) of the whole-exome region	WGS	68.2	60.8	70.9	66.1	63.5
Number of covered exome genes	WGS	20,915	20,083	21,148	21,378	20,747
Number of available exome genes for haplotyping	WGS	20,198	20,014	20,945		
Coverage (%) of the OMIM region	WGS	67.9	54.6	63.2	65.1	65.2
Number of covered OMIM genes	WGS	14,071	13,342	14,391	14,108	14,167
Number of available OMIM genes for haplotyping	WGS	13,512	12,377	13,710		
FPR (%)	WGS	30.22	26.9	—	28.9	28.6
	Targeted sequencing	31.7	30.4	—	32.9	28.7
ADO (%)	WGS	17.3	18.5	—	17.8	19.6
	Targeted sequencing	20.6	16.1	—	20.4	21.3
SNPs ∼4 Mb	WGS	5175	5102	6931		
	Targeted sequencing	817	682	719		
Key SNPs ∼4 Mb in the upstream region	WGS	16	6	32		
	Targeted sequencing	4	2	3		
Key SNPs ∼4 Mb in the downstream region	WGS	7	7	35		
	Targeted sequencing	0	0	6		

Data of the healthy family are represented by results from gradient 4 (15×).

With respect to FPR and ADO, we studied CFC178 from the deafness family and CFC616 from the hemophilia family; the LVAS family was excluded because of the lack of an amniotic fluid sample or a fetal villi sample. Overall, the ADO and FPR with WGS (CFC178: 17.3% ADO, 30.22% FPR; CFC616: 18.5% ADO, 26.9% FPR) were better than those with targeted region sequencing (CFC178: 20.6% ADO, 31.7% FPR; CFC616: 16.1% ADO, 30.4% FPR), indicating that the WGS produced more reliable SNP typing.

After haplotype analysis, the number of SNPs ∼4 Mb (2-Mb region upstream and downstream) around the pathogenic gene locus identified with WGS (CFC178: 5175; CFC616: 5102; CFC111: 6931) was significantly higher than that identified by panel sequencing (CFC178: 817; CFC616: 682; CFC111: 719). Similarly, more key SNPs were identified upstream and downstream with WGS (CFC178: 16/7; CFC616: 6/7; CFC111: 32/35) than with panel sequencing (CFC178: 4/0; CFC616: 2/0; CFC111: 3/6). More importantly, the key SNPs in the WGS data covered both upstream and downstream of the gene, accurately predicting the haplotype and determining carrier status. However, for two of the three families, targeted sequencing failed to capture any key SNP downstream of the disease-causing genes; thus, the haplotypes of their single cells could not be accurately determined (see the Discussion section). In summary, the WGS method is superior to the targeted panel sequencing in terms of genome coverage of targeted regions, sequencing quality, and haplotype analysis.

### Global genetic risk estimation based on single-cell WGS

We focused on whole human exonic and OMIM regions in the single-cell WGS data to evaluate the possibility of estimating global genetic risk. In brief, the genome coverages in the whole exonic region were 68.2% (CFC178), 60.8% (CFC616), and 70.9% (CFC111) and those in the OMIM gene region were 67.9% (CFC178), 54.6% (CFC616), and 63.2% (CFC111) (Table 2). Furthermore, the number of exome genes covered by WGS was 20,915 (91.9%) for CFC178 from the deafness family, 20,083 (88.2%) for CFC616 from the hemophilia family, and 21,148 (92.9%) for CFC111 from the LVAS family. Among them, the number of genes that could be used for haplotype analysis was 20,198 (88.7%), 20,014 (87.9%), and 20,945 (92.0%), respectively. Regarding the OMIM genes, 14,071 (92.8%) were covered in the deafness family, 13,342 (87.8%) in the hemophilia family, and 14,319 (94.4%) in the LVAS family. Among them, 13,512 (89.1%), 12,377 (81.6%), and 13,710 (90.3%) could be used for haplotype analysis, respectively. These results indicate that haplotypes for most genes can be predicted by combining single-cell WGS and haplotype analysis.

## Discussion

Prenatal diagnosis is an effective and necessary method for better managing inherited diseases. Invasive prenatal diagnosis methods, for example, amniocentesis, are often risky to pregnant women and fetuses (Agarwal & Alfirevic, 2012; Akolekar et al, 2015). As a supplement or even an alternative to invasive prenatal diagnosis, NIPT is potentially a better candidate for prenatal screening and diagnostics (Lau et al, 2014). Previous NIPT mainly focused on predicting chromosomal diseases (Porreco et al, 2014; Minarik et al, 2015; Zhang et al, 2019). Given the advances in sequencing technologies and data analysis, recent cffDNA-based NIPT studies are shifting the focus toward monogenic diseases (Zhang et al, 2019). However, the performance accuracy of this method is compromised because of the intrinsic limitations of cffDNA (Spits & Sermon, 2009; Beaudet, 2016; Rabinowitz & Shomron, 2020). Thus, new technologies and algorithms are urgently needed to improve the noninvasive prenatal diagnosis of monogenic diseases.

In addition to the use of cffDNA for NIPT, the circulating fetal nucleated red blood cells and cTBs in the maternal blood contain pure and complete fetal genetic information and could also be used for prenatal diagnostics (Choolani et al, 2012; He et al, 2017; Vossaert et al, 2018; Vossaert et al, 2021). The feasibility of cbNIPT in the prenatal detection of monogenic diseases (e.g., cystic fibrosis [Jeppesen et al, 2021] and spinal muscular atrophy [Beroud et al, 2003]) and preimplantation genetic testing have been demonstrated by several groups (Chang et al, 2021; Toft et al, 2021). Because of the lack of interference from restrictive placental mosaicism, the initial experiments focused on the application of circulating fetal nucleated red blood cells (Bianchi et al, 2002). However, probably because of the low abundance or instability of FNRBCs, most studies failed to capture FNRBCs in early pregnancy; thus, follow-up studies were conducted on cTBs and showed that the maternal peripheral blood contains 1–6 cTBs/ml in the first trimester of pregnancy (Oosterwijk et al, 1998; Bianchi et al, 2002). Regarding placental mosaicism, studying more fetal cells with potentially different genotypes from each sample can promote the accuracy of monogenic disease diagnostics using cbNIPT (Breman et al, 2016; Vossaert et al, 2019). In the future, it will be necessary to increase the number of cTBs by improving the efficiency of cell capture to ensure accurate prenatal diagnosis. In addition, most previous cbNIPT studies only focused on chromosomal (Breman et al, 2016) and subchromosomal (Kolvraa et al, 2016) abnormalities, and it remains largely unknown whether cbNIPT can be widely used for the prenatal diagnosis of monogenic diseases.

In our previous study (Chang et al, 2021), we used a combination of known markers, such as cytokeratin as a positive marker and CD45 as a negative marker (Hatt et al, 2014), to label cTBs as target fetal cells. In addition, single-cell STR analysis confirmed the source of isolated cTBs, indicating the feasibility of isolating cTBs from maternal blood. To avoid potential amplification errors and ADO during WGA (Liu et al, 2018), we subsequently took advantage of targeted sequencing of a 67-gene panel combined with haplotype analysis to detect monogenic diseases. In all deafness and ichthyosis disease cases, we successfully determined the inherited haplotypes of the fetus. However, some genes in the panel cannot be parsed because of ADO. In addition, targeted panel sequencing requires prior knowledge of disease-causing genes and mutations, limiting its application for the global estimation of genetic risk.

In the current study, we further explored the possibility of combining WGS with haplotype analysis for better prenatal diagnostics. Our results again demonstrated that cTBs isolated from maternal peripheral blood are sufficient for downstream genetic analyses to diagnose monogenic diseases. In addition, WGS combined with haplotype analysis successfully determined the genotype of the pathogenic gene in a fetus based on captured CFCs. Comparing WGS and targeted panel sequencing showed that WGS is superior to the targeted sequencing approach we previously used in terms of genome coverage, the number of SNP sites covering ∼4 Mb, the ADO ratio, and the FPR. Most importantly, more key SNPs were present in the WGS data than in the targeted sequencing, and WGS covered regions both upstream and downstream of the gene. These data highlight the value of WGS for precise haplotyping and prenatal diagnostics.

Furthermore, we focused on exome and OMIM regions to investigate the feasibility of genome-wide genetic risk evaluation. In general, single-cell WGS data showed 60–70% genome coverage for the exome and OMIM regions. However, haplotype analysis could be performed for ∼90% of the exome or OMIM genes (the upstream and downstream regions contained at least one key SNP), highlighting the possibility of diagnosing most genetic risks by combining single-fetal WGS data with familial haplotypes. Similar to cffDNA-based NIPT, fetoplacental mosaicism could be a major confounding factor for accurate diagnosis (Mardy & Wapner, 2016; Hartwig et al, 2017; Van Opstal et al, 2020; Rosner et al, 2021; Vossaert et al, 2021). In addition, the current method requires the proband for the haplotyping analysis, which limits its application for some affected families. More efficient fetal cell capture, better analysis algorithms, and more clinic data are required before the application of cbNIPT in prenatal testing or diagnostics. To the best of our knowledge, this is the first study to investigate the application of WGS from single captured cTBs in the prenatal diagnosis of monogenic disease and to estimate genetic risk on a whole-genome level. Overall, our study provides a novel and feasible NGS-based cbNIPT solution for targeted and global estimations of prenatal health. In the future, more clinical studies are required to investigate the feasibility of this method in diagnosing various genetic abnormalities and comprehensively evaluating fetal health.

## Materials and Methods

### Patient recruitment

The patient recruitment criteria were as follows: age between 20 and 45 yr; body mass index in the range of 18–25 kg/m^2^; 11–16 wk pregnant; family or one of the parents having a disease-causing genetic mutation, and the proband with the disease-causing mutation has been characterized (preferred). Women with one or more of the following conditions were excluded: fetus died in the uterus before sampling, no signed informed consent documentation, incomplete sampling, requested to withdraw from the study, and other circumstances that may affect the test results. A pregnant woman from a healthy family was also recruited as a control. This study was approved by the Scientific Research Ethical Committee of Peking University Third Hospital (approval reference number 2019-246-02). All subjects participating in the project signed an informed consent form.

### Samples collection

Maternal peripheral blood (15 ml) was drawn during pregnancy at 11–16 wk with an LBgard Blood Tube (Biomatrica) for fetal trophoblast cell capture and gDNA extraction. Amniotic fluid was collected for fetal chorionic cell capture and validation if applicable. Buccal swabs were taken at least three months after birth for gDNA extraction. Peripheral blood (4 ml in an EDTA anticoagulant tube) from the spouse and/or proband (if applicable) was collected for gDNA extraction.

### cTB enrichment and isolation

**Figure S3.**
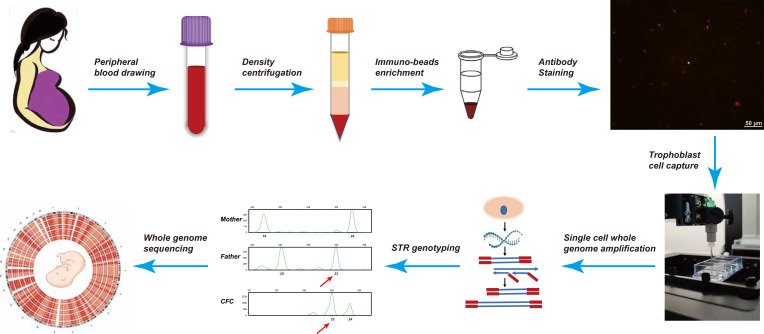
Flow chart of circulating trophoblast cells captured from maternal peripheral blood and characterization. Peripheral blood was obtained from pregnant women between the 11th and 16th gestational cycles, followed by centrifugation with lymphocyte separation medium to collect the nucleated cells. All nucleated cells were then enriched and stained to acquire candidate cTBs that met the selection criteria. Subsequently, the origin of candidate circulating trophoblast cells was identified by STR analysis after single-cell WGA. Finally, DNA libraries of all confirmed cTBs were sequenced and analyzed.

cTB enrichment and isolation were conducted, as previously described (Chang et al, 2021). Briefly, nucleated cells were collected by centrifugation with lymphocyte separation medium (density 1.077) after maternal blood collection. Subsequently, all nucleated cells were enriched using a cocktail of bead-linked antibodies (CD105, CD141, and HLA-G). The enriched cells were then stained with fluorescently labeled antibodies including DAPI (422801; BioLegend), anti-cytokeratin FITC (clone C11 628608; BioLegend), and anti-CD45 PE (clone 2D1 368510; BioLegend). Finally, we analyzed and screened candidate trophoblast single cells that met the selection criteria (DAPI positive, keratin positive, CD45 negative) using the UniPicker instrument (Unimed Biotech) for the subsequent steps. The sample processing is illustrated in Fig S3.

### Whole-genome DNA amplification of single cells and QC process

A PicoPLEX WGA Kit (R300672; Takara Bio) was used to amplify the whole genome of captured single cells. The WGA products were purified with DNA Clean Beads (N411-02; VAHTS) and stored at −20°C for further analysis. ∼1.5 μg of DNA products (∼200–1,000 bp in length) were recovered after WGA. gDNA was extracted with 200 μl of blood from the parents using a QIAGEN DSP Blood Mini Kit (61104; QIAGEN) according to the manufacturer’s directions. A genotyping assay was performed using an AmpFlSTR Identifiler Plus Kit (4427368; Applied Biosystems) containing 13 of the required loci from the Combined DNA Index System and three additional loci (D2S1338, D19S433, and the amelogenin gender-determining marker) to confirm the fetal origin of the isolated single cells. Briefly, STR–PCR was performed with the WGA products and the gDNAs from the parents. The PCR products were then subjected to capillary electrophoresis using an Applied Biosystems 3130xl Genetic Analyzer, and the genotypes were analyzed by GeneMapper 3.2 analysis software.

### Single-cell targeted sequencing

Panel design and library preparation details are described in our previous study (Chang et al, 2021). Briefly, 2 μg DNA libraries were hybridized to the custom panel according to the manufacturer’s instructions (Twist). Captured DNA libraries were sequenced using 150-bp paired-end index sequencing on a Hiseq 2500 (Illumina) instrument per the manufacturer’s instructions.

### Alignment, SNP/indel calling, and sequencing quality assessment

Adapters were trimmed from raw reads using fastp (Chen et al, 2018), and the reads were aligned to the reference genome (Hg19) using Burrows–Wheeler alignment (Li & Durbin, 2009) with the default parameters. PCR duplications were removed by Picard (https://broadinstitute.github.io/picard/). After filtering low-quality reads with mapping quality <10 and base quality <15, we recalibrated the base qualities and performed the SNP calling using the Genome Analysis Toolkit (GATK; filtering criteria: coverage >10 and quality value >20) (McKenna et al, 2010). We used the sequencing results from the father, mother, and proband to construct informative SNPs and marked the loci that may be affected by ADO as non-key SNPs; the others were designated key SNPs. The ADO ratio and FPR were calculated for the single-cell sequencing data from WGA products. The ADO ratio was calculated by the following equation:

$$\text{ADO}\;\text{ratio}=\frac{\sum {\text{SNP}}_{d}}{\text{Total}\;\text{SNPs}},$$

where SNP is the allele for which the amniotic fluid sample is heterozygous, whereas the single-cell sample is homozygous.

The FP ratio was calculated by the following equation:

$$\text{False}\;\text{Positive}\;\text{ratio}=\frac{\text{FP}\;\left(\text{false}\;\text{positive}\right)}{\text{Total}\;\text{SNPs}},$$

where FP indicates the number of loci with different genotypes between the control and single-cell samples.

Panel region coverage was calculated using single-cell WGS data by the following equation:

$$\text{Panel}\;\text{coverage}=\frac{\sum \text{covered}\;\text{region}}{\text{Total}\;\text{panel}\;\text{region}}\;.$$

This formula applies to the 67-gene region and the WES and OMIM gene regions.

In addition, analyses of the genome coverage by WGS in the gene region, the number of covered genes (≥1 reads), and the number of genes that could be used for haplotype analysis (both upstream and downstream contain at least one key SNP) were also performed in the WES (Agilent v6) and OMIM regions (https://www.omim.org/, version 2020.05.25, with a total of 15,145 genes).

### Library construction and WGS

Single-cell WGA products and gDNAs were sheared with Covaris to obtain fragments ranging from 250–350 bp in size. Paired-end sequencing libraries were prepared with KAPA Hyper Prep Kit (KK8504; Kapa Biosystems) as described in the manufacturer’s protocol. Barcodes were introduced during index adapter ligation for multiplex sequencing. DNA libraries were measured with an Agilent 2100 bioanalyzer (Agilent) for insert size and quantified by Qubit.

DNA libraries were sequenced using 150-bp paired-end index sequencing on a Hiseq 2500 (Illumina) device according to the manufacturer’s instructions. For WGS data from single-cell WGA products and parental gDNA from the healthy families, a total of eight gradients of randomly subsampled reads at 1X, 5X, 10X, 15X, 20X, 25X, 30X, and 35X human genome coverage were used to estimate the effect of sequencing depth on further analysis. The appropriate sequencing depth was selected based on the depth, FPR, ADO, and coverage of the 67-gene panel region; coverage of the whole-exon region; and coverage of the OMIM gene region for WGS on captured fetal trophoblast cells from healthy families.

### Single-cell haplarithmisis

**Figure 3.**
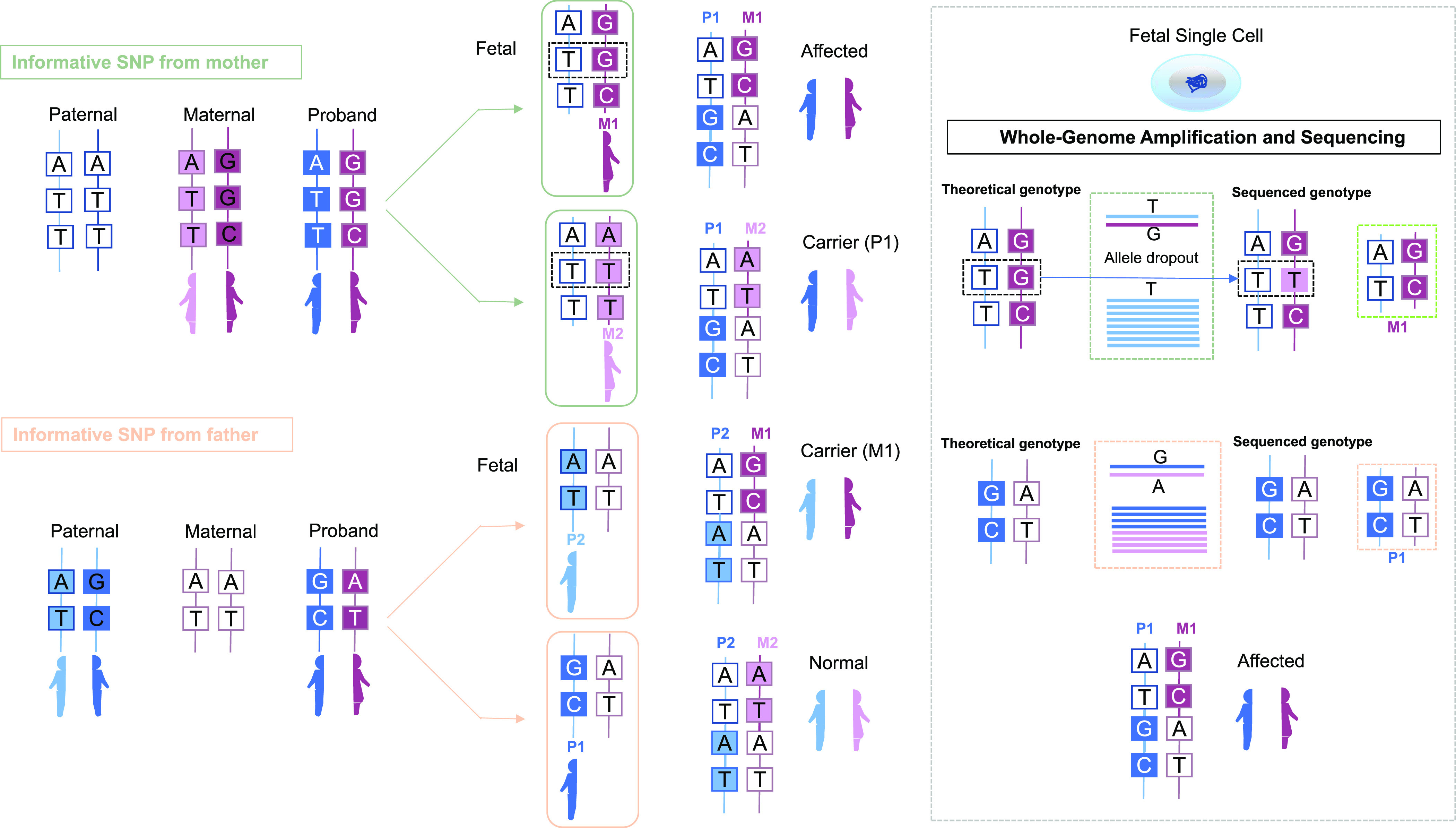
Workflow of embryonic haplotype construction using the genotypes of the father, mother, and proband. The details are described in the Materials and Methods section.

We combined the sequencing results of fetal trophoblast cells and the corresponding father, mother, and proband into a family for haplotype analysis (Handyside et al, 2010) (Fig 3). Assuming there is one homologous recombination on each chromosome inherited by the proband, the locus where the paternal genotype is AB and the maternal genotype is non-AB is IFF (informative SNP from father) and the locus where the maternal genotype is AB and the paternal genotype is non-AB is IFM (informative SNP from mother). We used the sequencing results of the father, mother, and proband to construct informative SNPs and marked the loci that may be affected by ADO as non-key SNPs, whereas the others were designated key SNPs. If the proband carried the haplotype of the paternal causative gene, it was marked as P1; otherwise, it was marked as P2. Similarly, if the proband carried the haplotype of the maternal causative gene, it was marked M1; otherwise, it was marked M2. Therefore, there are four possible combinations of haplotypes for the fetal trophoblast cells: P1/M1 (affected), P1/M2 (carrier from father), P2/M1 (carrier from mother), and P2/M2 (normal). Haplotype inheritance figures were drawn with all informative SNPs by matplotlib (https://github.com/matplotlib/matplotlib) according to the single-cell haplotype analysis results.

## Data Availability

The genome sequencing data for this publication have been deposited to the NCBI BioProject database (http://www.ncbi.nlm.nih.gov/bioproject/883017) under accession number PRJNA883017.

## Supplementary Material

Reviewer comments

## References

[bib1] Agarwal K, Alfirevic Z (2012) Pregnancy loss after chorionic villus sampling and genetic amniocentesis in twin pregnancies: A systematic review. Ultrasound Obstet Gynecol 40: 128–134. 10.1002/uog.10152 22125091

[bib2] Akolekar R, Beta J, Picciarelli G, Ogilvie C, D’Antonio F (2015) Procedure-related risk of miscarriage following amniocentesis and chorionic villus sampling: A systematic review and meta-analysis. Ultrasound Obstet Gynecol 45: 16–26. 10.1002/uog.14636 25042845

[bib3] Alfirevic Z, Navaratnam K, Mujezinovic F (2017) Amniocentesis and chorionic villus sampling for prenatal diagnosis. Cochrane database Syst Rev 9: CD003252. 10.1002/14651858.CD003252.pub2 28869276 PMC6483702

[bib4] Beaudet AL (2016) Using fetal cells for prenatal diagnosis: History and recent progress. Am J Med Genet C Semin Med Genet 172: 123–127. 10.1002/ajmg.c.31487 27133782

[bib5] Beroud C, Karliova M, Bonnefont JP, Benachi A, Munnich A, Dumez Y, Lacour B, Paterlini-Brechot P (2003) Prenatal diagnosis of spinal muscular atrophy by genetic analysis of circulating fetal cells. Lancet 361: 1013–1014. 10.1016/s0140-6736(03)12798-5 12660061

[bib6] Bianchi DW, Simpson JL, Jackson LG, Elias S, Holzgreve W, Evans MI, Dukes KA, Sullivan LM, Klinger KW, Bischoff FZ, (2002) Fetal gender and aneuploidy detection using fetal cells in maternal blood: Analysis of NIFTY I data. Prenat Diagn 22: 609–615. 10.1002/pd.347 12124698

[bib7] Brady P, Brison N, Van Den Bogaert K, de Ravel T, Peeters H, Van Esch H, Devriendt K, Legius E, Vermeesch JR (2016) Clinical implementation of nipt - technical and biological challenges. Clin Genet 89: 523–530. 10.1111/cge.12598 25867715

[bib8] Breman AM, Chow JC, U’Ren L, Normand EA, Qdaisat S, Zhao L, Henke DM, Chen R, Shaw CA, Jackson L, (2016) Evidence for feasibility of fetal trophoblastic cell-based noninvasive prenatal testing. Prenat Diagn 36: 1009–1019. 10.1002/pd.4924 27616633 PMC5129580

[bib9] Chang L, Zhu X, Li R, Wu H, Chen W, Chen J, Liu H, Li S, Liu P (2021) A novel method for noninvasive diagnosis of monogenic diseases from circulating fetal cells. Prenatal Diagn 41: 400–408. 10.1002/pd.5796 32673403

[bib10] Chen S, Zhou Y, Chen Y, Gu J (2018) Fastp: An ultra-fast all-in-one fastq preprocessor. Bioinformatics 34: i884–i890. 10.1093/bioinformatics/bty560 30423086 PMC6129281

[bib11] Choolani M, Mahyuddin AP, Hahn S (2012) The promise of fetal cells in maternal blood. Best Pract Res Clin Obstet Gynaecol 26: 655–667. 10.1016/j.bpobgyn.2012.06.008 22795236

[bib12] Clark AG (1990) Inference of haplotypes from pcr-amplified samples of diploid populations. Mol Biol Evol 7: 111–122. 10.1093/oxfordjournals.molbev.a040591 2108305

[bib13] Guo F, Wang D, Wang L (2018) Progressive approach for snp calling and haplotype assembly using single molecular sequencing data. Bioinformatics 34: 2012–2018. 10.1093/bioinformatics/bty059 29474523

[bib14] Handyside AH, Harton GL, Mariani B, Thornhill AR, Affara N, Shaw MA, Griffin DK (2010) Karyomapping: A universal method for genome wide analysis of genetic disease based on mapping crossovers between parental haplotypes. J Med Genet 47: 651–658. 10.1136/jmg.2009.069971 19858130

[bib15] Hartwig TS, Ambye L, Sorensen S, Jorgensen FS (2017) Discordant non-invasive prenatal testing (nipt) - a systematic review. Prenat Diagn 37: 527–539. 10.1002/pd.5049 28382695

[bib16] Hatt L, Brinch M, Singh R, Møller K, Lauridsen RH, Schlütter JM, Uldbjerg N, Christensen B, Kølvraa S (2014) A new marker set that identifies fetal cells in maternal circulation with high specificity. Prenat Diagn 34: 1066–1072. 10.1002/pd.4429 24912661

[bib17] He Z, Guo F, Feng C, Cai B, Lata JP, He R, Huang Q, Yu X, Rao L, Liu H, (2017) Fetal nucleated red blood cell analysis for non-invasive prenatal diagnostics using a nanostructure microchip. J Mater Chem B 5: 226–235. 10.1039/c6tb02558g 32263541

[bib18] Herzenberg LA, Bianchi DW, Schroder J, Cann HM, Iverson GM (1979) Fetal cells in the blood of pregnant women: Detection and enrichment by fluorescence-activated cell sorting. Proc Natl Acad Sci U S A 76: 1453–1455. 10.1073/pnas.76.3.1453 286330 PMC383270

[bib19] Hou S, Chen JF, Song M, Zhu Y, Jan YJ, Chen SH, Weng TH, Ling DA, Chen SF, Ro T, (2017) Imprinted nanovelcro microchips for isolation and characterization of circulating fetal trophoblasts: Toward noninvasive prenatal diagnostics. ACS Nano 11: 8167–8177. 10.1021/acsnano.7b03073 28721719 PMC5614709

[bib20] Jeppesen LD, Hatt L, Singh R, Ravn K, Kolvraa M, Schelde P, Uldbjerg N, Vogel I, Lildballe DL (2021) Cell-based non-invasive prenatal diagnosis in a pregnancy at risk of cystic fibrosis. Prenatal Diagn 41: 234–240. 10.1002/pd.5861 33150588

[bib21] Kolvraa S, Singh R, Normand EA, Qdaisat S, van den Veyver IB, Jackson L, Hatt L, Schelde P, Uldbjerg N, Vestergaard EM, (2016) Genome-wide copy number analysis on DNA from fetal cells isolated from the blood of pregnant women. Prenat Diagn 36: 1127–1134. 10.1002/pd.4948 27761919

[bib22] Kumar A, Ryan A, Kitzman JO, Wemmer N, Snyder MW, Sigurjonsson S, Lee C, Banjevic M, Zarutskie PW, Lewis AP, (2015) Whole genome prediction for preimplantation genetic diagnosis. Genome Med 7: 35. 10.1186/s13073-015-0160-4 26019723 PMC4445980

[bib23] Lau TK, Cheung SW, Lo PSS, Pursley AN, Chan MK, Jiang F, Zhang H, Wang W, Jong LFJ, Yuen OKC, (2014) Non-invasive prenatal testing for fetal chromosomal abnormalities by low-coverage whole-genome sequencing of maternal plasma DNA: Review of 1982 consecutive cases in a single center. Ultrasound Obstet Gynecol 43: 254–264. 10.1002/uog.13277 24339153

[bib24] Levy B, Stosic M (2019) Traditional prenatal diagnosis: Past to present. Methods Mol Biol 1885: 3–22. 10.1007/978-1-4939-8889-1_1 30506187

[bib25] Li H, Durbin R (2009) Fast and accurate short read alignment with burrows-wheeler transform. Bioinformatics 25: 1754–1760. 10.1093/bioinformatics/btp324 19451168 PMC2705234

[bib26] Liu W, Zhang H, Hu D, Lu S, Sun X (2018) The performance of malbac and mda methods in the identification of concurrent mutations and aneuploidy screening to diagnose beta-thalassaemia disorders at the single- and multiple-cell levels. J Clin Lab Anal 32: e22267. 10.1002/jcla.22267 28548214 PMC6817139

[bib27] Mardy A, Wapner RJ (2016) Confined placental mosaicism and its impact on confirmation of nipt results. Am J Med Genet Part C Semin Med Genet 172: 118–122. 10.1002/ajmg.c.31505 27184347

[bib28] McKenna A, Hanna M, Banks E, Sivachenko A, Cibulskis K, Kernytsky A, Garimella K, Altshuler D, Gabriel S, Daly M, (2010) The genome analysis toolkit: A mapreduce framework for analyzing next-generation DNA sequencing data. Genome Res 20: 1297–1303. 10.1101/gr.107524.110 20644199 PMC2928508

[bib29] Minarik G, Repiska G, Hyblova M, Nagyova E, Soltys K, Budis J, Duris F, Sysak R, Gerykova Bujalkova M, Vlkova-Izrael B, (2015) Utilization of benchtop next generation sequencing platforms ion torrent pgm and miseq in noninvasive prenatal testing for chromosome 21 trisomy and testing of impact of in silico and physical size selection on its analytical performance. PLoS One 10: e0144811. 10.1371/journal.pone.0144811 26669558 PMC4692262

[bib30] Nadler HL, Gerbie AB (1970) Role of amniocentesis in the intrauterine detection of genetic disorders. N Engl J Med 282: 596–599. 10.1056/NEJM197003122821105 4244215

[bib31] Oosterwijk JC, Mesker WE, Ouwerkerk-van Velzen MC, Knepfl CF, Wiesmeijer KC, Beverstock GC, van Ommen GJB, Kanhai HH, Tanke HJ (1998) Fetal cell detection in maternal blood: A study in 236 samples using erythroblast morphology, dab and hbf staining, and fish analysis. Cytometry 32: 178–185. 10.1002/(sici)1097-0320(19980701)32:3<178::aid-cyto3>3.0.co;2-g 9667506

[bib32] Palmer EE, Sachdev R, Macintosh R, Melo US, Mundlos S, Righetti S, Kandula T, Minoche AE, Puttick C, Gayevskiy V, (2021) Diagnostic yield of whole genome sequencing after nondiagnostic exome sequencing or gene panel in developmental and epileptic encephalopathies. Neurology 96: e1770–e1782. 10.1212/WNL.0000000000011655 33568551

[bib33] Porreco RP, Garite TJ, Maurel K, Marusiak B, Bombard A, Ehrich M, van den Boom D, Deciu C, Bombard A (2014) Noninvasive prenatal screening for fetal trisomies 21, 18, 13 and the common sex chromosome aneuploidies from maternal blood using massively parallel genomic sequencing of DNA. Am J Obstet Gynecol 211: 365.e1-365.e12. 10.1016/j.ajog.2014.03.042 24657131

[bib34] Rabinowitz T, Shomron N (2020) Genome-wide noninvasive prenatal diagnosis of monogenic disorders: Current and future trends. Comput Struct Biotechnol J 18: 2463–2470. 10.1016/j.csbj.2020.09.003 33005308 PMC7509788

[bib35] Rosner M, Kolbe T, Hengstschlager M (2021) Fetomaternal microchimerism and genetic diagnosis: On the origins of fetal cells and cell-free fetal DNA in the pregnant woman. Mutat Research/Reviews Mutat Res 788: 108399. 10.1016/j.mrrev.2021.108399 34893150

[bib36] Salomon LJ, Sotiriadis A, Wulff CB, Odibo A, Akolekar R (2019) Risk of miscarriage following amniocentesis or chorionic villus sampling: Systematic review of literature and updated meta-analysis. Ultrasound Obstet Gynecol 54: 442–451. 10.1002/uog.20353 31124209

[bib37] Satas G, Raphael BJ (2018) Haplotype phasing in single-cell DNA-sequencing data. Bioinformatics 34: i211–i217. 10.1093/bioinformatics/bty286 29950014 PMC6022575

[bib38] Simoni G, Brambati B, Danesino C, Rossella F, Terzoli GL, Ferrari M, Fraccaro M (1983) Efficient direct chromosome analyses and enzyme determinations from chorionic villi samples in the first trimester of pregnancy. Hum Genet 63: 349–357. 10.1007/BF00274761 6862440

[bib39] Simpson JL (2012) Invasive procedures for prenatal diagnosis: Any future left? Best Pract Res Clin Obstet Gynaecol 26: 625–638. 10.1016/j.bpobgyn.2012.05.007 22749621

[bib40] Singh R, Hatt L, Ravn K, Vogel I, Petersen OB, Uldbjerg N, Schelde P (2017) Fetal cells in maternal blood for prenatal diagnosis: A love story rekindled. Biomarkers Med 11: 705–710. 10.2217/bmm-2017-0055 28617034

[bib41] Spits C, Sermon K (2009) Pgd for monogenic disorders: Aspects of molecular biology. Prenat Diagn 29: 50–56. 10.1002/pd.2161 19101953

[bib42] Steele MW, Roy Breg W (1966) Chromosome analysis of human amniotic-fluid cells. Lancet 287: 383–385. 10.1016/s0140-6736(66)91387-0 4159775

[bib43] Toft CLF, Ingerslev HJ, Kesmodel US, Hatt L, Singh R, Ravn K, Nicolaisen BH, Christensen IB, Kolvraa M, Jeppesen LD, (2021) Cell-based non-invasive prenatal testing for monogenic disorders: Confirmation of unaffected fetuses following preimplantation genetic testing. J Assist Reprod Genet 38: 1959–1970. 10.1007/s10815-021-02104-5 33677749 PMC8417213

[bib44] Van Opstal D, Eggenhuizen GM, Joosten M, Diderich K, Govaerts L, Galjaard RJ, Go A, Knapen M, Boter M, Cheung WY, (2020) Noninvasive prenatal testing as compared to chorionic villus sampling is more sensitive for the detection of confined placental mosaicism involving the cytotrophoblast. Prenatal Diagn 40: 1338–1342. 10.1002/pd.5766 PMC7540368 32533714

[bib45] Volozonoka L, Miskova A, Gailite L (2022) Whole genome amplification in preimplantation genetic testing in the era of massively parallel sequencing. Int J Mol Sci 23: 4819. 10.3390/ijms23094819 35563216 PMC9102663

[bib46] Vossaert L, Wang Q, Salman R, Zhuo X, Qu C, Henke D, Seubert R, Chow J, U’Ren L, Enright B, (2018) Reliable detection of subchromosomal deletions and duplications using cell-based noninvasive prenatal testing. Prenatal Diagn 38: 1069–1078. 10.1002/pd.5377 PMC6587831 30357877

[bib47] Vossaert L, Wang Q, Salman R, McCombs AK, Patel V, Qu C, Mancini MA, Edwards DP, Malovannaya A, Liu P, (2019) Validation studies for single circulating trophoblast genetic testing as a form of noninvasive prenatal diagnosis. Am J Hum Genet 105: 1262–1273. 10.1016/j.ajhg.2019.11.004 31785788 PMC6904821

[bib48] Vossaert L, Chakchouk I, Zemet R, Van den Veyver IB (2021) Overview and recent developments in cell-based noninvasive prenatal testing. Prenatal Diagn 41: 1202–1214. 10.1002/pd.5957 PMC9355411 33974713

[bib49] Walknowska J, Conte FA, Grumbach MM (1969) Practical and theoretical implications of fetal-maternal lymphocyte transfer. Lancet 293: 1119–1122. 10.1016/s0140-6736(69)91642-0 4181601

[bib50] WHO | human genomics global health (2019) Available at: http://www.who.int/

[bib51] Zhang CZ, Adalsteinsson VA, Francis J, Cornils H, Jung J, Maire C, Ligon KL, Meyerson M, Love JC (2015) Calibrating genomic and allelic coverage bias in single-cell sequencing. Nat Commun 6: 6822. 10.1038/ncomms7822 25879913 PMC4922254

[bib52] Zhang J, Li J, Saucier JB, Feng Y, Jiang Y, Sinson J, McCombs AK, Schmitt ES, Peacock S, Chen S, (2019) Non-invasive prenatal sequencing for multiple mendelian monogenic disorders using circulating cell-free fetal DNA. Nat Med 25: 439–447. 10.1038/s41591-018-0334-x 30692697

[bib53] Zipursky A, Hull A, White FD, Israels LG (1959) Foetal erythrocytes in the maternal circulation. Lancet 1: 451–452. 10.1016/s0140-6736(59)92264-0 13632052

